# Lambda gp*P*-DnaB Helicase Sequestration and gp*P*-RpoB Associated Effects: On Screens for Auxotrophs, Selection for Rif^R^, Toxicity, Mutagenicity, Plasmid Curing

**DOI:** 10.3390/v8060172

**Published:** 2016-06-22

**Authors:** Sidney Hayes, Wen Wang, Karthic Rajamanickam, Audrey Chu, Anirban Banerjee, Connie Hayes

**Affiliations:** Department of Microbiology and Immunology, College of Medicine, University of Saskatchewan, Saskatoon, SK S7N 5E5, Canada; wen.wang@me.com (W.W.); kar029@mail.usask.ca (K.R.); audreymchu@gmail.com (A.C.); abanerj7@uwo.ca (A.B.); clh127@outlook.com (C.H.)

**Keywords:** bacteriophage lambda (λ) replication initiation protein P, *E. coli* DnaB replicative helicase, Replicative Killing phenotype, *rpoB* encoding β-subunit of RNA polymerase (RNAP), *rpoB* mutations suppressing P-lethality, ColE1 plasmid curing, screening for auxotrophs, selecting for Rif^R^ mutants, P-DnaB sequestration, cellular mutagenesis

## Abstract

The bacteriophage lambda replication initiation protein P exhibits a toxic effect on its *Escherichia coli* (*E. coli*) host, likely due to the formation of a dead-end P-DnaB complex, sequestering the replicative DnaB helicase from further activity. Intracellular expression of P triggers SOS-independent cellular filamentation and rapidly cures resident ColE1 plasmids. The toxicity of P is suppressed by alleles of *P* or *dnaB*. We asked whether P buildup within a cell can influence *E. coli* replication fidelity. The influence of P expression from a defective prophage, or when cloned and expressed from a plasmid was examined by screening for auxotrophic mutants, or by selection for rifampicin resistant (Rif^R^) cells acquiring mutations within the *rpoB* gene encoding the β-subunit of RNA polymerase (RNAP), nine of which proved unique. Using fluctuation assays, we show that the intracellular expression of P evokes a mutator effect. Most of the Rif^R^ mutants remained P^S^ and localized to the Rif binding pocket in RNAP, but a subset acquired a P^R^ phenotype, lost sensitivity to ColE1 plasmid curing, and localized outside of the pocket. One P^R^ mutation was identical to rpo*Q148P, which alleviates the UV-sensitivity of *ruv* strains defective in the migration and resolution of Holliday junctions and destabilizes stalled RNAP elongation complexes. The results suggest that P-DnaB sequestration is mutagenic and supports an earlier observation that P can interact with RNAP.

## 1. Introduction

**Figure 1:**
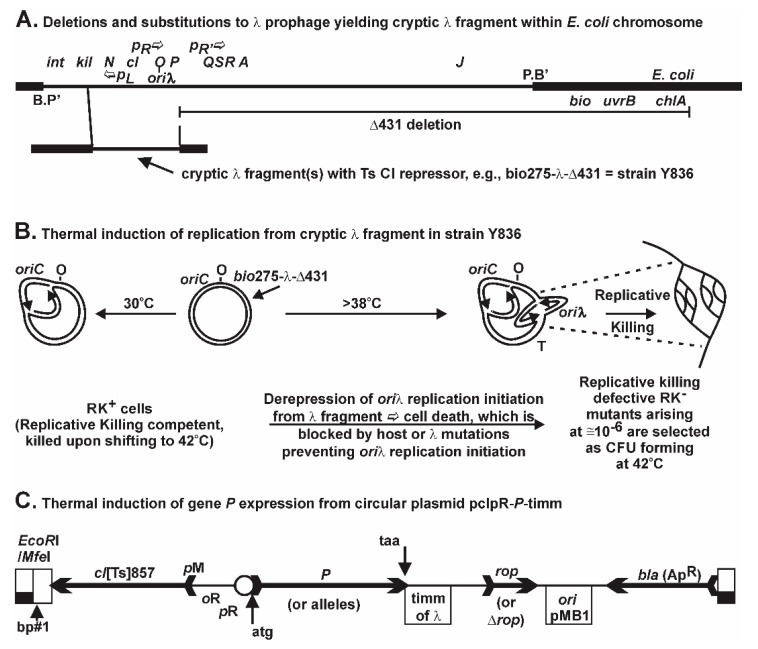
Modes for expression of *P*: (**A**) cryptic prophage map (for more λ gene detail refer to Figure 1B in reference [35] or Figure 1 in reference [36]); (**B**) λ gene expression is induced by shifting cells from 30 °C to 42 °C, which inactivates the encoded Ts (temperature sensitive) *cI*[Ts]857 repressor, permitting rightward transcription from promoter *p*R; and (**C**) an exact copy of *P* (or alleles of *P*) was cloned into the synthetic expression plasmid pcIpR-timm [35] (shown by arrows at ATG and TAA). *P* expression from pcIpR-*P*-timm is controlled by the λ CI[Ts] repressor. The circle to the right of *pR* and left of ATG represents the ribosomal binding site for the deleted intervening gene *cro*. The transcription of *P* from the plasmid terminates at the transcriptional terminator *timm*, which in the wild type λ sequence prevents both the low maintenance and high level establishment modes of *cI-rexA-rexB* transcription from transcribing leftward from *p*M or *p*E into *o*L*-p*L [37].

A lambda (λ) prophage is maintained within the chromosome of *Escherichia coli* (*E. coli*) by an action of the CI repressor encoded by gene *cI*. The inhibition of replication initiation from the prophage *ori*λ site positioned midway within gene *O* [1,2,3] (Figure 1A) is blocked by CI protein binding to operator sites overlapping promoters directing transcription leftward from *p*L, e.g., copying genes *N-int*, or rightward from *p*R for expressing genes *cro-cII-O-P-Q*. The product of gene *P* (gp*P*, P) participates in loading the *E. coli* DnaB helicase [4] onto DNA during formation of a DnaB:P:O:*ori*λ preprimosomal complex [5,6,7], each factor being required for replication initiation from *ori*λ. DnaB unwinds double-stranded (ds) DNA at the replication fork, by encircling and translocating along the 5′ lagging strand using energy provided by ATP hydrolysis [4]. The interaction between DnaG primase and the N-terminal end of DnaB [8] increases both the NTPase and helicase activities of DnaB and the synthesis of RNA primers by DnaG [4,8,9,10,11,12,13,14,15]. DnaB can promote the progression of Holliday junctions, believed important in the repair of DNA damage occurring near advancing replication forks [16,17]. DnaB functions as a hexamer, with about 20 hexamers per cell [18,19], with each binding up to six ATP [20,21]. It forms a complex with the host replication initiation protein DnaC to which the majority of DnaB in a cell is bound [22]. This interferes with the intrinsic single-stranded (ss) DNA binding activity of DnaB [20,22,23,24,25,26]. The DnaC-DnaB complex acquires cryptic ssDNA binding activity specific to the bacterial origin of replication, *oriC*, where it begins unwinding dsDNA after a number of complex changes [27,28,29,30,31,32,33,34].

The λ P protein has evolved to compete for and dissociate DnaC-DnaB complexes [5,38] and two to six P monomers can bind to every DnaB hexamer [5,39,40], forming an enzymatically inactive “dead-end” complex [22]. High levels of P were found deleterious to host cells [41,42] and it was suggested that P sequestered, *i.e.*, bound-up, DnaB and thus interfered with the initiation of host DNA synthesis. ColE1 replication establishment or propagation [43] is extremely sensitive to P [35]. The effect is suppressed by alleles of *dnaB* or *P*, which suggests that P can impact a cellular replication event by acting outside of the DnaA and *oriC*-dependent replication initiation step. This same study showed that P-dependent cellular filamentation arose in cells defective for SOS induction, suggesting that P was influencing replication propagation or restart mechanisms. For example, the sequestration of DnaB by P could perturb or impede origin-independent stable DNA replication [44,45,46,47,48], where both cellular replication restart and ColE1 replication depend upon PriA helicase activity [49,50,51,52] at R-loops.

**Figure 2:**
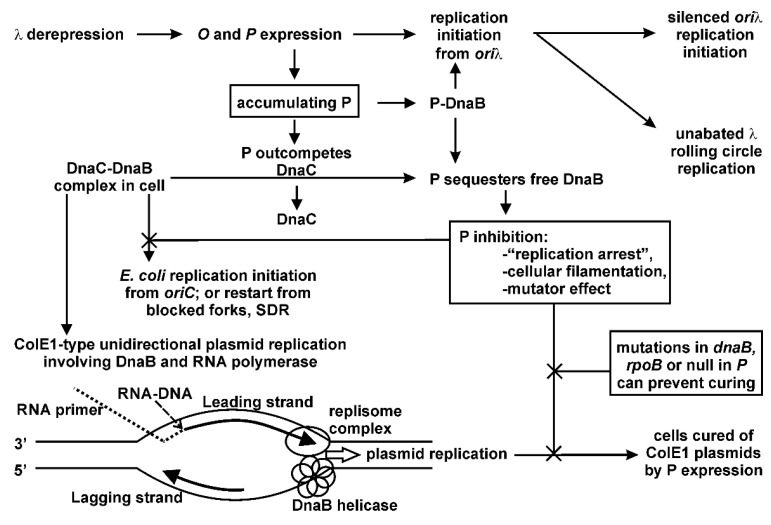
Summary of P lethality/inhibition observations, including observations provided herein. Arrows terminating in X show inhibitory activities. “SDR” is stable DNA replication [44,45,46,47,48]. P can bind DnaB helicase and replace pre-bound DnaC.

Multiple possibilities present for the P-lethality/inhibition observations, and are summarized in Figure 2. The 233-amino acid P protein is very stable, with a half-life of an hour [53,54]. In addition to binding Oλ [55], P interacts with the host proteins DnaA [56,57,58], DnaB [59,60,61], GrpE [62], DnaJ and DnaK [63], and possibly RNA polymerase (RNAP) [64]. As is understood from *in vitro* studies, the Hsp70 chaperone complex removes P from DnaB when the P-DnaB complex is bound to *ori*λ:O [40] in a two-step reaction involving DnaJ binding to complexed P-DnaB, which enhances P-DnaK binding, ATP hydrolysis, and the release of a P-DnaK-ADP complex. There is a requirement for a high concentration of DnaK unless GrpE is present, and a proposal that DnaK changes the conformation of P from a native to a folded state that is no longer able to bind DnaB [65]. An existing hypothesis for P-lethality/inhibition is that by binding up DnaB (or DnaA), P will inhibit chromosomal replication initiation from *oriC*. Greater understanding of the extent of intracellular binding between P and DnaB, which occurs beyond that involved in the formation of an *ori*λ preprimosomal complex is required to help explain DnaB sequestration. We showed that: (a) very low levels of P are necessary for the limited *ori*λ initiation required for λ replication [35]; and (b) that λ replication resulting in high phage burst from an induced λ*cI*[Ts]857 *S*am7 prophage blocked for cell lysis occurred in cells that were simultaneously fully derepressed for *P* expression from pcIpR-*P*-timm—conditions where DnaB would be sequestered.

The initiation of *ori*λ replication from a chromosomally integrated, but non-excisable λ gene fragment (Figure 1A) is repressed by the *cI* [Ts] repressor. When CI[Ts] is inactivated upon shifting cells growing at 30 °C to 42 °C, *O-P* are transcribed, replication initiation arises at *ori*λ, and the Replicative Killing, RK^+^, phenotype is triggered, resulting in massive cell death [66]. Rare mutations nullifying the RK^+^ phenotype permit the selection for colony forming units (CFU) at 42 °C, each representing RK^−^ mutants in host or prophage genes (not merely in *P*) that block some aspect of λ replication initiation [66,67,68,69]. For example, we characterized within RK^−^ mutants small deletions in *O* that shift the reading frame and yield close-by stop codons polar for downstream gene expression. These RK^−^ mutants form CFU at 42 °C, likely surviving by limiting downstream *P* expression. Thus, it appears these induced mutants can tolerate some level of λ *P* expression (or that of any other toxic λ fragment protein, such as the unstable *cII* gene product). Since P expression had been shown to be toxic, confusion existed relative to its contribution to the RK^+^ phenotype. We suggested the terms *cis* Replicative Killing and *trans* P-lethality to distinguish mechanistically these two ideas. We found that the RK^+^ phenotype was uniquely dependent upon multiple, non-repairable λ replication forks arising from *ori*λ within a trapped prophage fragment, resulting in very rapid, nonreversible cell death, whereas there are likely multiple possibilities for P-lethality, which can be reversed even after hours of *P* expression [35]. In short, limited levels of P can be metabolized. The *dnaB*grpD55 allele [70], which encodes missense mutations V256I and E426K [35] was shown to fully prevent *ori*λ replication initiation and to suppress cell killing by 10^6^-fold from the lambda prophage fragment in strain Y836 (Figure 2C in reference [71]) when the cells were shifted from 30 °C to 42 °C, even though *P* expression remained constitutive from the induced defective prophage. This allele was shown Ts for λ replication but not for *E. coli* cell growth [70]. When removing the complication of the defective prophage and simply expressing *P* from a plasmid, we found that the same allele of *dnaB* suppressed the various P-lethality phenotypic manifestations [35]. This suggested that the P-lethality phenotype was dependent upon a P-DnaB interaction, possibly DnaB sequestration, and required further study.

In order to test whether P-DnaB interactions influence cellular replication events around the chromosome, rather than just at *oriC*, we explored a hypothesis that P buildup within a cell can perturb host replication fidelity. Since blocks to replication restart can arise around the chromosome, we first surveyed for an increase in the appearance of auxotrophic mutations of any type, and then for a targeted increase in rifampicin resistant (Rif^R^) mutations arising in *rpoB*. The assumption made was that perturbing replication restart can be error prone. Herein, we observed a λ-dependent mutator effect that was linked to *P*. We observed that *P* expression creates the potential for sequestration of DnaB and results in a dramatic increase in cellular mutagenesis, which is nullified by inactivated or altered alleles of *P* and by an allele of *dnaB*.

## 2. Results

### 2.1. Examination for Auxotrophs within RK^−^ Population

To determine if the events related to replication initiation from *ori*λ influence cellular mutation, we asked if the selected RK^−^ clones acquire additional untargeted mutations within the chromosome. Since there are hundreds of host genes involved in cell metabolism [72], representing a very large genetic target of perhaps 1/6th of the chromosome, we examined whether any of the RK^−^ clones derived from a prototrophic RK^+^ host acquired an auxotrophic phenotype. If mutations conferring auxotrophy arise during the selection for RK^−^ mutants, then individual RK^−^ clones will appear at higher frequency when their selection is plated on RM compared to MM where they cannot form a colony. Accordingly, the ratio of RK^−^ CFU arising on RM/MM should be greater than unity. Indeed, this was observed (refer to rightmost columns in Table 1 and Table 2).

In order to undertake this experiment we first prepared a His^+^ transductant of Y836 *his* (moved from host 594) (Figure 1A), and then moved the λ *cIII-ren* fragment from Y836 into prototrophic host strains 594 and W3101 to create 594::(*cIII-ren*)λ and W3101::(*cIII-ren*)λ. We next demonstrated that these RK^+^ strains and their parents plated with equal efficiency on rich (RM) and minimal (MM) media (Table S1) at 30 °C. Then, RK^−^ CFU from Y836 *his* and the Y836 His^+^ transductant were isolated on RM, MM, or MM supplemented with histidine, biotin, or Casamino acids (not vitamin-free), and the RK^−^ frequency was compared (Table 1). The appearance of RK^−^ CFU was equivalent on RM or Casamino acids-supplemented MM (shown to be equivalent to RM), but was lower on un-supplemented MM, which did not support the growth of many RK^−^ mutants acquiring an auxotrophic mutation. This suggested that auxotrophic mutations were co-selected during the selection for RK^−^ clones. Thirty-seven RK^−^ isolates from Y836 *his* were screened for their auxotrophic defect [73] using the stabbing technique of Holliday [74], but including histidine in all of the supplemented MM plates. Sixteen categories of auxotrophs were identified. Some were further characterized by reversion analysis and yielded revertants at frequencies between 9.5 × 10^−8^ and 1.3 × 10^−7^, suggesting that the auxotrophs had acquired missense mutations rather than deletions, which agreed with a parallel observation that a high proportion of the RK^−^ clones had acquired a Ts auxotrophic phenotype when plated on MM. Since there was no obvious difference between the original Y836 *his* grown on MM + histidine and the Y836 His^+^ transductant grown on MM, we continued to use the original Y836 *his* strain to avoid any unaccounted transduced traits moved into Y836 His^+^, and hereafter refer to Y836 *his* as Y836.

Is an intrinsic, λ-independent, mutator effect in Y836 cells responsible for the selection of auxotrophic markers within the selected RK^−^ CFU? RK^−^ CFU were selected on RM and MM using prototrophic 594 and W3101 cells into which the (*cIII-ren*)λ fragment was moved by transduction from Y836. The frequency of RK^−^ CFU from both 594::(*cIII-ren*)λ and W3101::(*cIII-ren*)λ cells was reduced on MM compared to that found on RM. The similarity of the results to those with Y836 cells suggested the effect was linked to the (*cIII-ren*)λ fragment and not to a “λ-independent” activity activated at 42 °C (Table 1). Y836 was made *lexA*3[Ind] or Δ*recA* to block SOS induction, or made *dinB* (DNA polymerase IV) or *umuCumuD* (DNA polymerase V) to prevent DNA damage tolerance [75]. Neither removing the capacity for SOS induction nor Pol IV or Pol V activities eliminated the appearance of auxotrophs within the selected RK^−^ CFU (Table 1). What is responsible for the RK^−^ frequency being higher on RM than on MM?

### 2.2. Hypothesis of P as Mutator, Evaluating Using Screen for Auxotrophy

Table 2 reveals that the RM/MM CFU ratio remains at unity for all strains where λ-fragment derepression was blocked by pCI^+^ but was elevated when *ori*λ replication initiation was blocked but transcription of the λ fragment was allowed at 42 °C. This suggests that the putative mutator effect accounting for the reduction in colony formation on MM plates at 42 °C is linked to the induction of gene expression from the (*cIII-ren*)λ fragment but that it does not require actual replication initiation from *ori*λ. The conclusions from Table 2 are limited to the suggestion that any of the inducible λ fragment gene products could be responsible for the increased recovery of auxotrophs on RM *vs.* MM. However, it is important to insert prior information that is relevant to this experiment: we previously found (Figure 2C in reference [71]) that the addition to strain Y836 of plasmid pCI^+^ expressing wild-type *imm*λ *cI* repressor, or the addition by transduction of the *dnaB*grpD55 allele, each prevented replication initiation and blocked Replicative Killing when cells with a λ prophage fragment were shifted from 30 °C to 42 °C. The plasmid pCI^+^ blocked both derepression of λ fragment transcription and replication initiation from *ori*λ. The *dnaB*grpD55 allele fully suppressed Replicative Killing of Y836 cells shifted from 30 °C to 42 °C (Figure 2C in [71]), even better than with the pCI^+^ plasmid, but did not block the induced expression of the λ fragment genes. In addition, we previously compared cell viability (Table 6 in reference [35]) for 594 *dnaB*grpD55[pcIpR-*P*-timm] cells grown up in culture at 25 °C by plating them at 25 °C (cell viability assumed 1.0), 37 °C, 39 °C (viabilities averaged 0.99), and 42 °C (viability 1.0). This showed that the *dnaB*grpD55 allele did not impart cellular toxicity over this temperature range, and that it could completely suppress the toxicity resulting from *P* expression from this plasmid. In comparison, 594[pcIpR-*P*-timm] cells had a viability of 0.001 when plated at 42 °C. Therefore, it can obliquely be argued from Table 2 that the increase in the RM/MM CFU ratio for the *dnaB*grpD55 cells is more complex than simply equating it to a manifestation of cell toxicity.

To examine the suggestion that λ gene expression could contribute to the mutator effect, a stab assay was evoked to screen for the proportion of acquired auxotrophic mutations within selected RK^−^ CFU, Table 3. This permitted a direct analysis of the linkage and potential co-selection between prophage induction and the appearance of auxotrophs within selected RK^−^ mutants. Using strain Y836 *his* as an example, the frequency of RK^−^ CFU selected at 42 °C on RM (for ten independent selections, all values × 10^−6^) was: 16.9, 11.2, 13.5, 16.5, 2, 3.5, 1.1, 5.6, 1.3, and 0.9. Individual CFU arising on RM plates incubated at 30 °C were stabbed to MM plates incubated at 30 °C to check whether any spontaneous auxotrophs arose within the starting cells. Similarly, *all* the selected CFU (large or tiny) arising per RM plate(s) that were incubated at 42 °C and yielded ~10 to ~100 CFU/selection plate were picked with sterile toothpicks onto two MM+Histidine plates. One stab plate was incubated at 30 °C and another at 42 °C to determine if the CFU possessed a Ts auxotrophic mutation, and a parallel set were stabbed to an RM plate incubated at 30 °C to ensure transfer of cells had occurred. All stab plates were incubated for 48 h. Potential auxotrophs were picked from the RM control plate and streaked onto MM for auxotrophy confirmation. Stabs for RK^−^ mutants from Y836 were also made to MM+His+biotin plates, which allowed us to demonstrate that the drop in RK^−^ frequency on MM relative to RM was not frequently linked to loss of the *bio* operon introduced by the *bio*275 addition (Figure 1A), which if deleted along with the λ fragment would confer a Bio^−^ phenotype. In summary, among CFU arising at 30 °C or 42 °C from strains without the (*cIII-ren*)λ fragment, or where the fragment was blocked for λ gene expression by pCI^+^, less than 0.01% were auxotrophs. In contrast, for strains with the (*cIII-ren*)λ fragment that were derepressed for λ gene expression, about 3.4% of the CFU forming at 42 °C were auxotrophs. No auxotrophs were observed among the stabbed CFU when the Y836 strain was engineered to contain a partial gene replacement substituting Kan^R^ within *P*, or if *P* was inactivated as in the RK^−^ mutant 566a with a spontaneous IS*2* insertion in *P*. The observation that cell growth, with concomitant λ gene *P* expression, elevates the frequency of CFU acquiring an auxotrophic phenotype suggests the hypothesis that *P* expression confers a mutator phenotype in growing cells.

### 2.3. P Expression Stimulates Selection for Rifampicin-Resistant (Rif^R^) CFU

To examine if the influence of *P* expression could be duplicated using a different scheme, we switched from screening for auxotrophs to a direct forward selection for rifampicin resistant (Rif^R^) CFU. The influence of *P* expression was measured in cells derepressed for the (*cIII-ren*)λ fragment, or 594 cells transformed with plasmid pcIpR-*P*-timm (Figure 1C) in Table 4. When *P* is expressed in cells with a *dnaB*^+^ allele there is an enormous increase in the number of selected Rif^R^ CFU. Inactivation of *P* by insertion, deletion, or alteration by a point mutation (*P*^π39991^) nullified the mutator effect of P expression, as did replacing *dnaB*^+^ with the *dnaB*grpD55 allele. Both the prophage and plasmid data suggest that a P-DnaB interaction is involved in the P-mutator phenotype.

When the RK^+^ phenotype is induced (*i.e.*, combining *cis*-killing with *trans*-P-lethality) the frequency of Rif^R^ mutants was stimulated by >20-fold over situations with *trans*-P-lethality, but lacking *cis*-killing. For example, in Table 4: divide the Rif^R^ stimulation factors 88,727, 42,500, or 51,485 for the *O*^+^ *P*^+^ RK^+^ strains Y836 and 594::(*cIII*-*cI*857-*O*^+^-*P*^+^-*ren*)λ by the factor of 2167 for the *P*^+^ strain defective in *O*, *i.e.*, Y836 *O*223a *P*^+^. Mutant *O*223a carries a deletion in *O* resulting in a frameshift, and a premature nonsense codon that may evoke polarity for the expression of downstream *P*. The 20-fold enhancement in *rpoB* mutagenesis when the RK^+^ phenotype is induced requires further explanation. Do higher levels of P yield progressively higher mutagenic consequences? Additionally, further properties of the DnaBgrpD55 protein, *i.e.*, beyond its ability to block *ori*λ replication initiation [71] and to nullify P- lethality [35], remain to be explored.

Two fluctuation assays (FA) were employed to determine if the Rif^R^ mutations arising in *rpoB* preexisted the induction of *P* expression. In FA#1, we grew up 40 FA tubes of cells for 26–27 generations to saturation at 25 °C (without any *P* expression) [77]. From each of the 40 tubes, parallel aliquots were removed (each aliquot representing 1−2 × 10^8^ CFU) and spread on RM+100 µg/mL rifampicin plates (Table 5). One plate from the split aliquots was incubated at 25 °C and the other at 37 °C (which permitted partial expression of *P* from the plasmid). The 80 incubation plates yielded between 0 CFU (observed for 45 plates) to 11 Rif^R^ CFU per plate. Aliquots taken from 23 of the 40 tubes yielded Rif^R^ CFU at either 25 °C or 37 °C, or on both plates. Thirty individual Rif^R^ CFU were subcloned and the *rpoB* gene from each mutant was sequenced. Eighteen of these CFU were selected from the 25 °C plates and 12 CFU were from the 37 °C plates. Twenty distinct *rpoB* mutation sites were identified within the 30 Rif^R^ CFU. One mutation (1691, P564L) occurred four times, four arose two times, and the remainder were separate mutational events. Only one FA tube yielded the same *rpoB* mutation (P564L) for the parallel 25 °C and 37 °C selections. Because different mutations occurred in the cells incubated at 37 °C compared to their counterparts at 25 °C in almost all cases, and because very few Rif^R^ colonies were recovered on each plate, the mutations have most likely occurred long after the culture was split into two aliquots and spread, *i.e.*, the Rif^R^ mutations mostly arose during growth on the spread plates, where one set of cells was exposed to some P (plates incubated at 37 °C) and the other set was not (plates incubated at 25 °C).

In FA#1 we also asked if there was a toxic effect of P expression at 37 °C that influenced the appearance of Rif^R^ mutants. Several of the 40 FA#1 culture tubes from the 48 h incubation at 25 °C were diluted in buffer and spread on RM agar plates (without rifampicin) that were incubated at 25 °C or 37 °C. The titers for cells from culture tubes that were incubated on RM plates at 25 °C were ~2 × 10^9^ CFU/mL. The titers for the same diluted cells that were incubated on RM plates at 37 °C were on average 82-fold less, showing the toxicity of low level *P* expression from the plasmid at 37 °C (*i.e.*, where *P* was not fully induced), when compared to growth on plates at 25 °C where *P* expression from the plasmid was repressed. Clearly, the Rif^R^ CFU arising on the 37 °C plates in FA#1 were exposed to 82-fold cellular P-toxicity. The results suggest that P triggers a mutagenic effect resulting in an increase in Rif^R^ mutations, and that some of the Rif^R^ CFU arising on the 37 °C Rif^100^ plates can resist/survive P toxicity. In FA#2 and subsequent experiments, we examined the influence of P on the selection of Rif^R^ CFU in order to help address the possibilities that Rif^R^ mutants selected in the presence of P are resistant to P-toxicity and that P triggers mutagenesis.

**Figure 3:**
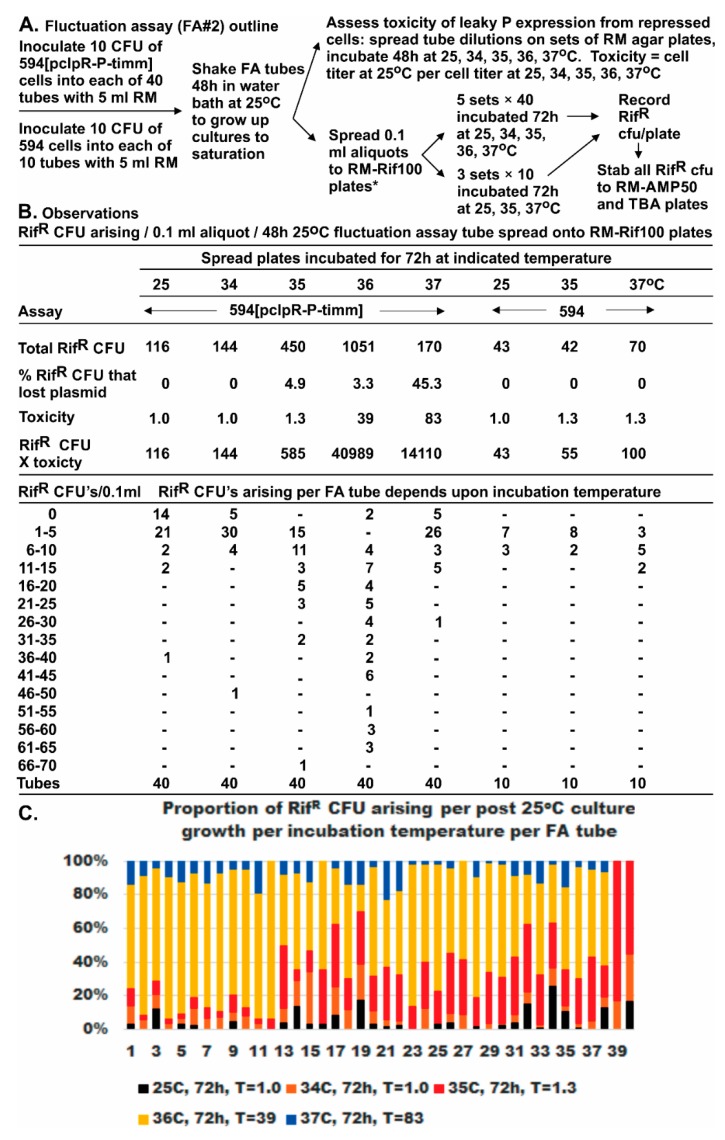
Fluctuation assay to examine the influence of *P* expression on the screen for Rif^R^ CFU: (**A**) outline of methodology; (**B**) observations; and (**C**) relative proportion of Rif^R^ CFU arising on spread plates incubated in parallel at 25 °C to 37 °C, where T = the toxicity of the treatment, determined by CFU formed on RM plates without rifampicin at 25 °C per CFU formed on RM plates without rifampicin after incubation at 25, 34, 35, 36, or 37 °C.

In FA#2, it was possible to superimpose the influence of *P* expression toxicity on 40 populations of 594[pcIpR-*P*-timm] cells derived from cultures each grown to saturation at 25 °C and then spread on parallel plates that were incubated for 72 h at higher temperatures (Figure 3). We show that the Rif^R^ CFU were increased on the parallel Rif^100^ plates incubated at 35–36 °C (Figure 3B,C). A nine-fold increase in Rif^R^ CFU arising on the Rif^100^ plates incubated at 36 °C (Figure 3B) was seen even though the incubation reduced the viability of the cells spread from the 25 °C growth tubes by 39-fold. Full derepression of *P* from pcIpR-*P*-timm occurs between 39 °C and 42 °C, but at lower temperatures some CI[Ts] repressor activity remains to bind *o*R and limit *P* expression from *p*R [35]. The partially induced *P* expression at 36 °C from pcIpR-*P*-timm is both toxic and mutagenic. The result shows that many of the beneficial mutations conferring Rif^R^ arise after imposing the P-expression selection rather than being preexisting, which supports the formation of a mutagenic state resulting from P accumulation. The increased expression of *P* in cells incubated at 37 °C caused 83-fold cell lethality. This reduced the selected Rif^R^ CFU by six-fold compared to incubation at 36 °C (Figure 3B) suggesting that most of the acquired Rif^R^ *rpoB* mutations do not confer resistance to P-lethality.

### 2.4. Assessing if Selecting Rif^R^ CFU co-Selects for P^R^ Cells

**Figure 4:**
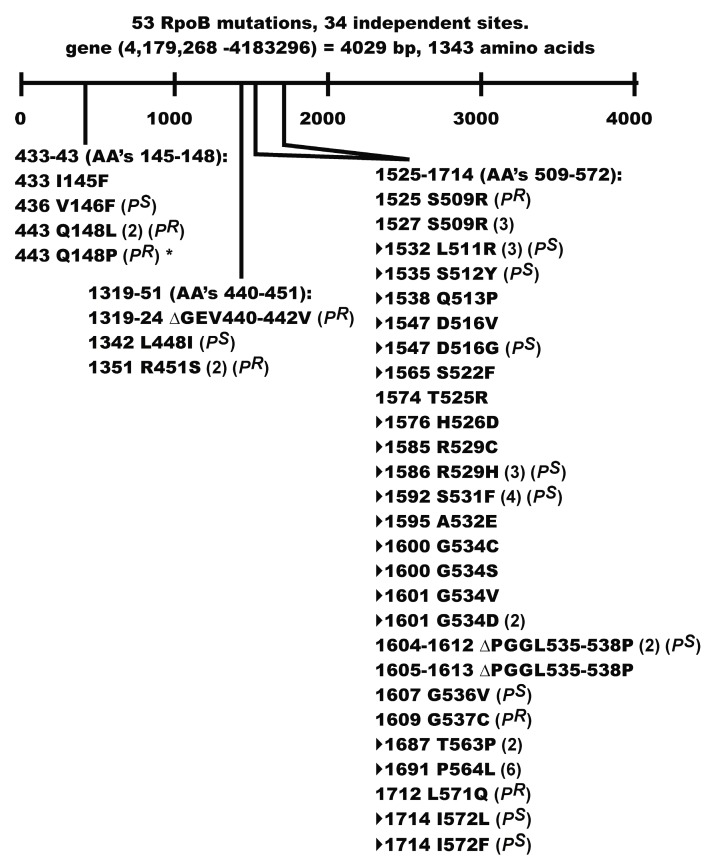
Location of selected Rif^R^ mutations in *rpoB*. Mutants designated *P*^S^ are transformable at 30 °C but not 37 °C by pcIpR-*P*-timm, whereas the *P*^R^ mutants were transformable at both temperatures. The *rpoB* mutant designated with * is identical to mutant rpo*148 (see Discussion). The missense mutations (designated by sideways triangle) each fall within codons for amino acids found in the Rif binding pocket of *rpoB*.

Cellular sensitivity to *P* expression from pcIpR-*P*-timm was demonstrated [35] by showing that sensitive cells are transformable at 25–30 °C, where the CI[Ts] repressor is active, but not at 37 °C where the CI[Ts] repressor activity is reduced, permitting some *P* expression. Two categories of Rif^R^ mutants were screened in Table 6, those from cells where P had never been introduced into the cell (Table S2), and survivors from FA#1 that were exposed to P, had lost pcIpR-*P*-timm during their growth on plates at 37 °C, and had survived a 82-fold toxic effect of P-exposure. Two of the 11 Rif^R^ CFU selected without cell exposure to P showed weak P^R^ and the remainder were P^S^. Six of the 11 Rif^R^ CFU arising from the FA#1 incubation at 37 °C varied from being high to moderately P^R^, revealing that some Rif^R^ mutations confer P^R^, while most others are P^S^ (Figure 4).

**Figure 5:**
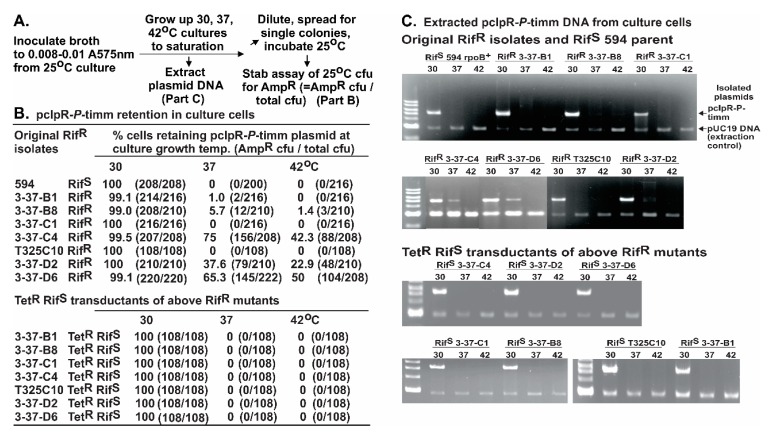
pcIpR-*P*-timm retention in P^S^ and P^R^ Rif^R^ isolates and for Rif^S^ transductants. (**A**) Outline of experimental method is identical to that described for measuring *P*-induced plasmid loss in Figure 3, reference [35]: Cultures made from single CFU for each isolate grown up on Amp^50^ plates were inoculated and grown to stationary phase in RM plus 50 µg/ml ampicillin for 48 h at 25 °C. Cell aliquots from the cultures were diluted into fresh RM (no ampicillin) as shown in outline A and incubated for about 20 h in shaking baths between 30 to 42 °C. (**B**) The percentage of the Tet^S^ Rif^R^ cells that acquired the Tet^R^ Rif^S^ phenotypes after transduction (see text) was 81%, 85%, 75%, 94%, 69%, 88%, and 90%, for the Rif^R^ mutants B1, B8, C1, C4, C10, D2, and D6, respectively. (**C**) Extracted plasmids from identical culture cells (described in **A**, quantitated in **B**) grown between 30 to 42 °C.

Table S3 shows 109 characterized mutations within *rpoB* conferring Rif^R^. Figure 4 summarizes 53 Rif^R^ mutations we localized to 34 independent sites within *rpoB*, representing 25 mutations that were previously identified, and 9 unique mutations including base pair sites (designated by ##, Table S3) 433(I145F), 1351(R451S), 1532(L511R), 1595(A532E), 1600(G534S), 1609(G537C) and the deletions 1319-1324(ΔGEV440-442V), 1604-1612(ΔPGGL535-538P), and 1605-1613(ΔPGGL535-538P). One previous deletion in *rpoB* conferring Rif^R^, ∆1589-97 base pairs, was described [78]. The 109 Rif^R^ mutations localize to five general regions of *rpoB* (expanded from Figure 1 of [79]): region I, codons 139-153(181); II, codons 395-451; III, codons 507-574 (major group with 79 of 109 mutations; previously subdivided into three groups [79]); IV, codons 631-687; and V, codons 1244-1260. Structural studies of *Thermus aquaticus* RNA polymerase with Rif revealed that Rif binds and is inhibited through the same biochemical mechanism as for *E. coli* RNAP [79]. These authors identified deep within the main DNA/RNA channel a Rif binding pocket in RpoB (Figure 5 in reference [79]), which for *E. coli* represents amino acids (clockwise circular orientation) 511, 510, 512, 513, 514, 515, 526, 516, 518, 522, 521, 686, 563, 564, 573, 529, 572, 531, 532, 533, and 534. Thirty-four of the 52 *rpoB* mutations we sequenced were missense mutations within codons for these amino acids (Figure 4). The two deletions ∆PGGL535-538P (Figure 4) fell just outside of this pocket. The seven P^R^ mutations (Figure 4) Q148L, Q148P, ∆*GEV440-442V*, *R451S*, S509R, G537C, and *L571Q* (italicized showing strong P^R^) fell outside of the pocket and may define independent contact points between RpoB and P.

### 2.5. Sensitivity of ColE1 Plasmid Replication to P-inhibition

Culture cells with a *grpD55* allele of *dnaB* have a P^R^ phenotype and are refractory to curing of the ColE1-based pcIpR-*P*-timm plasmid when grown at 37 °C or 42 °C [35], whereas P^S^ *dnaB*^+^ culture cells are cured of pcIpR-*P*-timm when incubated at or above 35 °C. The influence of Rif^R^ mutations on P-dependent ColE1 curing is shown in Figure 5. The Rif^R^ mutants were transformed with pcIpR-*P*-timm and examined for retention of the plasmid at 30 °C, 37 °C and 42 °C. The P^S^ Rif^R^ mutants 3-37-C1 and T325C10 were cured of pcIpR-*P*-timm during cell growth at 37 °C or 42 °C as was the Rif^S^ 594 parent. The remaining P^R^ Rif^R^ mutants 3-37-B1, -B8, -C4, -D2 and -D6 showed varying levels of plasmid retention at 37 °C and 42 °C. These mutants were sequenced through *dnaA* to determine if they possessed an *rpl* (resistance to P-lethality) mutation shown to map within *dnaA* [56]. Each P^R^ Rif^R^ mutant was wild type for *dnaA*. The results show that some alleles of *rpoB*, *i.e.*, the same ones conferring P^R^, can suppress ColE1 plasmid curing by P.

The original non-transformed Rif^R^ mutants (shown left column, Figure 5) were transduced to Rif^s^ by P1 transduction of Tet^R^ Rif^S^ from donor cells. The Tet^R^ transductants were screened for replacement of the Rif^R^ *rpoB* allele with a Rif^S^ *rpoB*^+^ allele and loss of the Rif^R^ phenotype. Each of the Tet^R^ Rif^S^ transductants were sequenced for *rpoB* and shown to have lost the *rpoB* mutation originally conferring the Rif^R^ phenotype.

All of the sequenced Tet^R^ Rif^S^ transductants were then transformed with pcIpR-*P*-timm at 25 °C and then examined for plasmid loss after culture cell growth at 30, 37, or 42 °C. Each of the Tet^R^ Rif^S^ transductants retained the pcIpR-*P*-timm plasmid when grown at 30, but completely lost the plasmid during culture growth at 37 °C or 42 °C, Figure 5. These results show that replacement of the Rif^R^ allele with the *rpoB*^+^ allele restores the sensitivity of ColE1 to P.

The sequenced P^R^ Rif^R^ isolates and their Tet^R^ Rif^S^ transductants (see Figure 5) were also transformed with pcIpR-*P*-timmΔ*rop* to increase selective pressure from the higher copy plasmid (Table 7 and Figure S1). These results further support the results in Figure 5 showing that some Rif^R^ *rpoB* alleles, especially those that show a P^R^ phenotype, interfere with the sensitivity of ColE1 to P. 

## 3. Discussion

The initiation of replication from a trapped λ fragment (Figure 1B) results in backward, or wrong-orientation replication forks that can be problematic for cell viability [44,48,80,81]. The leftward fork from *ori*λ can undergo head-on collisions with the rightward replication fork arising from *oriC*, or with rightward-directed transcription arising, e.g., from five of the seven *rrn* operons: *rrnC*, *A*, *B*, *E*, and *H*, positioned between *oriC* and *ori*λ. Such head-on collisions are likely responsible for the powerful RK^+^ phenotype. We previously compared the kinetics of cell death resulting from *P* expression from pcIpR-*P*-timm or Replicative Killing by initiation from a trapped *ori*λ [35]. The *cis*-RK^+^ phenotype became irreversible by 20 min while *trans*-P lethality/inhibition was slower and reversible for several hours. The RK^+^ phenotype (Figure 1B) seems uniquely dependent upon unresolved collisions between replication arising from *ori*λ and the rightward fork from *oriC* or with cellular transcripts. Of course, when the trapped λ fragment is induced, both *cis*-RK^+^ and *trans*-P-lethality/inhibition phenotypes are jointly produced. Normal λ induction would likely escape the *cis*-RK^+^ phenotype because the prophage excises from the chromosome, which is not possible for the defective prophage. The combined lethal effects may amplify the selective pressure for survivors and be responsible for the 20-fold increase in mutants arising compared to situations where only *P* is expressed. This extreme selective pressure may account for the very high frequency of co-selected auxotrophs among the selected RK^−^ mutants, especially for the RK^−^ CFU acquiring a mutation conferring Ts auxotrophy. For example, any co-selected Ts auxotrophic mutation arising in an amino acid synthesis or utilization pathway would inhibit protein synthesis at the limiting temperature and reduce λ fragment gene expression, in turn lowering the buildup of P, and help such RK^−^ mutants be selected for colony formation during 42 °C selection period. As previously noted, RK^−^ mutants arising on RM at 42 °C are defective for *ori*λ replication and can form a CFU at 42 °C. Any host mutation that facilitates colony formation during cell growth at 42 °C could be co-selected. While never previously explored in the literature, co-selected Ts auxotrophs might be particularly supportive in situations where the RK^−^ mutation does not inactivate *P*, for example mutations nullifying *O* activity. However, none of the original characterized *O*-defective *P*^+^ RK^−^ starting strains employed herein had acquired an auxotrophic mutation when originally isolated (and all were then maintained at non-inducing temperatures to prevent further selective pressure). Nevertheless, a high proportion of auxotrophs appeared from these strains when re-isolated CFU were obtained from 42 °C plates when *P*^+^ was expressed. Given the subsequent results suggesting that P conferred a mutator effect, we explain the high proportion of auxotrophs (refer to Table 3 section “Induced defective λ lysogens”) as representing a combination of P mutagenesis combined with a powerful unrealized selection for cellular mutations that limited *P* expression toxicity during cell growth at 42 °C. It is also entirely possible that some of the Rif^R^ *rpoB* CFU selected at 37 °C (cells with plasmid) or 42 °C served to reduce cellular toxicity to *P* expression. Further investigation is required to explore potential manifestations of P toxicity on alternative mutation selection; however, this may simply be an inherent limitation in all studies where a mutagenic agent is also toxic, and where toxicity can influence selection. One possibility is that the selected mutants are resistant to the toxic agent. We explored this difficulty in FA#2 by accounting for both toxicity and recovered Rif^R^ mutants for each selection temperature. We could demonstrate that many Rif^R^ mutants were sensitive to P-toxicity, and, for selections at 36 °C, which were less toxic than those at 37 °C, there was a significant increase in the appearance of Rif^R^ mutant CFU (see below). A separate manuscript is in preparation on the complementation and examined toxicity of cloned *vs.* defective prophage (single copy) expressed λ *imm*-*rep* gene products.

Fluctuation assays were used to determine if the Rif^R^ mutations arising in *rpoB* preexisted the expression of *P*. As noted, this was complicated by observations that 80-fold cellular toxicity occurred in cells with pcIpR-*P*-timm incubated at 37 °C, and that most of the Rif^R^ CFU were P^S^. On lowering the incubation temperature to 36 °C, P-lethality was reduced by half but the recovered Rif^R^ mutants increased by nine-fold compared to 30 °C. With incubation lowered yet another degree to 35 °C, the toxicity of P was minimal (1.3-fold), ColE1 curing of cells was slight (5%), yet the Rif^R^ mutants recovered were 3.9-fold higher than at 30 °C. These observations suggest that the increase in Rif^R^ mutants was dependent upon P expression, rather than selection conditions favoring preexisting P^R^ Rif^R^ mutants.

Seven of the Rif^R^ mutants acquired a P^R^ phenotype. Two of these mutations, G537C and L571Q map close to the Rif binding pocket in RpoB [79], but the remaining mutations Q148L, Q148P, ∆GEV440-442V, R451S, and S509R fell outside of the pocket. These mutations may help define a contact point(s) between RpoB and P that has long been suggested [64]. Our P^R^ mutation Q148P in *rpoB* is identical to rpo*148 [82]. A small collection of rpo* mutations, which possess some level of Rif^R^ [82], were isolated based on the discovery [83] that mutations in RNAP alleviate the UV-sensitive phenotype of *ruv* strains, e.g., rpo*148 suppresses the extreme sensitivity of UV treatment to a ∆*relA* ∆*spoT ruv* strain. This property was linked to the rpo* mutation destabilizing RNAP open complexes and stalled elongation complexes, thereby reducing the occurrence of stalled RNAP(s) at lesions in the DNA template [82,83]. Co-directional collisions between the replisome and RNAP in an arrested (backtracked) elongation complex can lead to DNA double strand breaks [84]. Transcription pausing and stalling regulators include ppGpp which destabilizes open complexes, and the RNAP modulators DskA, GreA, GreB, and Mfd [81,84,85]. The failure of rpo* RNAP to pause and backtrack, and their ability to reduce the accumulation of RNAP arrays [81], helps explain why rpo* mutations can suppress the formation of DSB [84]. Assuming that our P^R^ Q148P Rif^R^ mutation will reduce the accumulation of RNAP arrays, can this explain a P^R^ phenotype and the suppression of P-dependent ColE1 plasmid curing? Future studies require determining if the other P^R^ mutants share an rpo* phenotype.

Several unanswered problems arise in relation to the observed mutator effect caused by *P* expression. Based on the low λ requirement for P, coupled with P sequestration of the low cellular amount of DnaB [18,19], either *P* expression from λ requires a high degree of negative regulation to prevent P-lethality/inhibition (probably not fully appreciated), or there is an unknown mechanism for limiting the P^S^ phenotype. The apparent complete escape of λ replication from P is contrasted with the exquisite sensitivity of ColE1 plasmid replication to P, and its mutagenic effect on *E. coli*. One possible explanation is that late λ rolling circle replication may be independent of replication restart. Whereas, the mechanism for initiation of ColE1 [43] is similar to restart (see [35]), and likely requires loading DnaB for each initiation event, which breaks down with P-DnaB sequestration. Just how P^R^ Rif^R^ mutants suppress ColE1 curing by P, and seemingly overcome P-DnaB sequestration remain open questions.

The NH_2_-terminal portion of P was suggested to bind O and its C-terminal domain to interact with host proteins [86,87]. The NH_2_-terminal domain of DnaC is involved in binding to DnaB. Both P and DnaC share a stretch of amino acids with high homology at their NH_2_ termini [88]. The in-frame ∆76 deletion within codons 9–86 at the NH_2_-terminal end of P suppressed: (a) the curing of ColE1 plasmids; (b) P-lethality at 37–39 °C [35]; and (c) prevented a P-dependent increase in Rif^R^ mutations or mutagenic effect. These observations suggest that the NH_2_ end of P can compete for the DnaC binding site on DnaB, and that P binding at this site can account for P^S^ phenotype: its cellular toxicity, ColE1 curing, and mutator activity.

The cell construct, Figure 1A, possessed a mutagenic activity that was linked to the expression of *P* from the λ fragment within the chromosome. Independently, cells that included a plasmid where *P* was the only inducible gene product exhibited a mutator phenotype when *P* was expressed. The mutator activity was assessed by screening for auxotrophic mutations arising during Replicative Killing selection for RK^−^ clones, or the selection of Rif^R^ mutations within *rpoB*. Since alleles of the host *dnaB* replicative helicase, or of *P*, can nullify the mutator phenotype, and since P interacts with DnaB to form an enzymatically inactive “dead-end” P-DnaB complex [22], *i.e.*, the so-called sequestered DnaB, reducing or eliminating cellular DnaB activity is linked to the mutator phenotype.

Under conditions of DnaB sequestration, any replication fork collapse could be problematic for replication re-initiation and generate gaps in DNA synthesis needing repair. Studies reported with yeast suggested that the stalling of transcription at abasic (AP) sites is highly mutagenic [89] and mechanisms, such as mutations arising via translesion synthesis through AP sites were proposed. Most of the auxotrophs we characterized acquired temperature sensitive mutations, characteristic of a missense arising from a point mutation. We did not find that blocking SOS induction, deleting *recA*, or inactivating individual *E. coli* mutator polymerases Pol IV or Pol V eliminated P-dependent mutagenesis. Since three *E. coli* DNA polymerases, Pol II, Pol IV and Pol V, are involved in induced mutagenesis [90], this was not evaluated rigorously as the lack of any one could be substituted for by another. Further work is needed to determine if the sequestration of DnaB by P stimulates the appearance of gaps and AP sites around the chromosome, linking them to the mutagenic state associated with elevated levels of P. This would agree with the observation that P can stimulate SOS-independent filamentation [35]. We propose a model that P sequestration of DnaB has several cellular outcomes: (a) it prevents the loading of DnaB helicase needed for the initiation of ColE1 replication (Figure 2) resulting in rapid, complete plasmid curing; (b) it prevents replication restart requiring the reloading of DnaB by DnaC; and (c) when a faster-moving Pol III replisome complex inevitably collides with RNAP, the displaced replisome components along with RNAP from the leading strand are uncoupled from DNA polymerase copying the lagging strand, leading to ssDNA regions that become a target for DNA damage which increase the probability for spontaneous mutation. We speculate that P may exacerbate this situation by gaining access to DnaB remaining bound to the lagging strand, and so interfere with rebinding of the β-clamp and Pol III, resulting in replication stuttering and the formation of leading strand gap(s) as illustrated for the co-directional collisions drawn in Figure 5e of reference [91]. We imagine that mutations in RNAP that limit P-interaction, or limit arrested RNAP complex formation on DNA, hence reducing collisions between the replisome and arrested RNAP, will lessen the influence of P-lethality/inhibition.

## 4. Materials and Methods

### 4.1. Strains Employed

The bacteria and plasmids employed are listed in Table 8 or in reference [35].

### 4.2. Oligonucleotides Employed for PCR Fragment Amplification and DNA Sequencing

The DNA oligonucleotides employed are shown in Table S4.

### 4.3. Growth Medium, Buffers, PCR Amplification, DNA Sequencing, and Fluctuation Assays

Solid support growth medium designated RM (rich medium) includes 10 g Bacto agar, 10 g Bacto tryptone and 5 g of NaCl per liter. MM (minimal medium) includes 11 g Bacto agar, 100 mL of 10× M9 salts (70 g Na_2_HPO_4_, 30 g KH_2_PO_4_, 5 g NaCl, 10 g NH_4_Cl in 1 L deionized water), 16 mL 25% glucose, 0.1 mL 1 M CaCl_2_, 1 mL 1 M MgSO_4_·7H_2_O, and 0.2 mL 0.3% ferric citrate per liter [98]. The broth versions of RM and MM omit agar. MM agar plates were, when noted, supplemented with histidine (100 µg/mL), biotin (1 µg/mL), or 0.3% Bacto casamino acids (not vitamin-free), and adjusted to 0.01 M Tris-HCl, pH 7.6. Ampicillin (Amp), rifampicin (Rif), or tetracycline (Tet) were added to RM or MM agar preparations at 50, 100, or 15 µg/mL, respectively. MM agar plates were made up for auxotroph typing using the combinations of amino acids (each at 0.1 mg/mL), vitamins and purine/pyrimidine pools (concentration/mL) as per Holliday [74]: adenine (0.05 mg/mL), hypoxanthine (0.05 mg/mL), folic acid (0.5 µg/mL), ornithine (0.1 mg/mL), pantothenic acid (0.5 µg/mL), guanine (0.05 mg/mL), pyridoxine (0.5 µg/mL), sodium thiosulfate (0.05 mg/mL), thymine (0.01 µg/mL), p-aminobenzoic acid (0.5 µg/mL), uracil (0.05 mg/mL), riboflavin (2.5 µg/mL), nicotinic acid (0.5 µg/mL), choline (10 µg/mL), inositol (5 µg/mL), and biotin (0.01 µg/mL). Plates containing Rif were held in foil covers and discarded after a week without use. The growth medium employed for transformation and transduction was LB (10 g Bacto tryptone, 5 g Bacto yeast extract, 5 g NaCl per liter), and for electroporation was SOB {2% w/v Bacto tryptone, 0.5% yeast extract, 10 mM NaCl, plus Mg^2+^ (2 mL 1 M MgCl_2_·6H_2_O and 2 mL 1 M MgSO_4_·7H_2_O per 200 mL SOB)}, and for SOC was SOB + Mg^2+^ made 1.8 µg/mL with 25% glucose. The Φ80 buffer used for cell dilutions, and the buffers for DNA handling, plasmid purification, and electrophoresis, and the methods for PCR fragment amplification and for DNA sequencing were previously described [76]. The methodology employed for fluctuation assays #1 and #2 is described in Table 5 and Figure 2, respectively.

### 4.4. Transformation, Transduction, Electroporation, and Recombineering

Procedure for transduction: P1vir lysates were prepared on strains with a Tet^R^ marker (Table 8); this was repeated to prepare a secondary lysate grown up on the same host. All the lysates employed had titers of 5 × 10^8^ PFU/mL or higher. A single colony of a Tet^S^ Rif^R^ *E. coli* mutant was grown up overnight at 30 °C in LB broth; 5.0 mL of these cells were centrifuged at 6 K rpm for 6 min, decanted, and the cell pellet resuspended in an equal volume of sterile MC buffer (0.1 M MgSO_4_, 0.005 M CaCl_2_). The resuspended cells were aerated for 15 min in a shaking water bath at 30 °C. Cells-phage mixtures and mock infections were incubated at 37 °C for 20 min to permit phage adsorption, then an equal volume of sterile 0.1 M citrate buffer (0.1 M citric acid, pH 5.5) was added to prevent the re-adsorption of P1 phage. The mixtures were added to 7.8 mL of pre-warmed LB broth + 0.2 mL of 0.1 M citrate buffer, and then incubated at 37 °C in a shaking water bath for 60–90 min. The samples were pelleted at 6 K for 10 min, washed, and the cell pellet was resuspended in 400 µL of 0.1 M Citrate buffer. One hundred microliters aliquots were spread on agar plates with 15 µg/mL of tetracycline and incubated at 30 °C overnight. The Tet^R^ transduced single colonies arising were streaked onto fresh tetracycline and rifampicin plates to identify the transduced Tet^R^ Rif^S^ and Tet^R^ Rif^R^ CFU.

The procedure used for transformation, was from reference [35]. For electroporation and recombineering we followed the procedures outlined in reference [96].

### 4.5. Moving Chromosomal Fragments by PI and Complementation for P

Every P1*vir* lysate was serially passaged twice on the donor strain to avoid any carry-over of markers before being used to move gene fragments into recipient cells. Strain CAG12147 *nadA*57::Tn*10* possesses a tetracycline resistance (Tet^R^) marker at 16.85 min on the *E. coli* linkage map. Tet^R^ was moved into Y836 *his*^+^. A second step was to co-transduce the Tet^R^ marker plus the cryptic λ prophage genes (*cIII-ren*)λ into two recipient prototrophic strains 594 and W3101. Tet^R^ recipients were screened for the *imm*λ fragment using the functional immunity (FI) recombination assay [71]. Strain CAG12164 has a *malF*::Tn*10* Tet^R^ marker at ~91.5 min. The Tet^R^ marker was moved by transduction into DE407 *lexA*3[Ind] recipient and the Tet^R^ CFU were examined for increased sensitivity to UV to confirm that they retained the *lexA*3[Ind] allele. Both Tet^R^ and *lexA*3[Ind] alleles were transduced from DE407*malF*::Tn*10* into recipient strain Y836, which represents *bio*275[substituting for *int-kil*]-*cIII*-*ren*-∆431 (Figure 1A). The Tet^R^ CFU were screened for acquisition of *lexA*3[Ind] by assaying for increased UV sensitivity. Note that the *bio*275 addition in Y836 confers UvrB^+^ and Bio^+^ phenotypes to cells with deletion ∆431. The presence of *imm*λ phenotype was confirmed by the CFU being lysed by λ*vir* and resistant to λ*cI*72 at 30 °C. Strain SF2006 has a *dinB*::Tn*5* at 5 min and can form CFU on plates with kanamycin. A similar approach was used to introduce *umuC*112:Tn*5* into strain Y836. The ethylmethanesulfonate (EMS) spot assay was performed on the Kan^R^ transductants to verify movement of the *dinB* allele into Y836. Kan^R^ transductants were grown overnight, washed twice and 0.1 mL aliquots and spread onto MM+His plates in quadruplet. Two of the four plates were spotted with 5 µL concentrations of EMS, incubated 48 h at 30 °C, and compared. Cells defective for DinB mutator polymerase gave fewer auxotrophs in region of EMS spot (compared to DinB^+^ cells) as the concentration of EMS was increased. The *rpoB*^+^ gene was moved by P1*vir* transduction from Tet^R^ donor strain CAG18500 *thiC*:Tn*10* into Rif^R^ isolates of 594. Tet^R^ CFU were isolated and Tet^R^ Rif^S^ co-transductants (occurring at about 80%) were distinguished by stabbing on agar plates containing 100 µg/mL of rifampicin.

The phage λ*imm*434 *P*am3 carries the amber mutation CAG(Gln)39786TAG and can only form plaques on host cells that can complement for P, or on a host with a nonsense suppressor mutation, as TC600 *supE*. For complementation assays the phage is diluted into Φ80 buffer and aliquots were mixed with 0.3 mL of assayed host cells plus top agar and poured onto RM plates incubated between 25 °C to 42 °C. The efficiency of plating (EOP) for assay conditions is compared to parallel plates where TC600 is the permissive host (assumed to give a %EPO of 100) and 594 the non-permissive host, upon which *P*^+^-revertant phages arise at a frequency of about 1 × 10^−7^. Complementation for *P* expression from plasmid pcIpR-*P*-timm was described in reference [35]. Some examples include (%EOP at 38–39 °C/30 °C): Y836 (65/0.05), Y836 ilr 534c (41/0.02), Y836 ilr *O*208b (67/0.001), Y836 ilr*O*223a (35/0.008), and Y836 ilr *P*::IS*2* (0.0002/0.00008). “ilr” represents a characterized λ mutation conferring “initiation of λ replication” defective phenotype.

## Figures and Tables

**Table 1:** RK^−^ mutation frequencies.

RK^+^ Strains	RK^−^ CFU Frequency on RM ×10^−6^ (SE × 10^−6^) ^a^	RK^−^ CFU Frequency on MM ×10^−6^ (SE × 10^−6^) ^b^	Supplement to MM ^b^	RK^−^ CFU at 42 °C Arising on RM/MM ^c^
**RK^−^ mutant frequencies determined from selection at 42 °C on RM and MM ^a^.**				
Y836 *his* ^d^	5.88 (1.0)	0.373 (0.057)	histidine	15.8
Y836 *his*	5.0	0.33	histidine + biotin	15.1
Y836 *his* ^e^	4.05 (0.3)	6.08 (0.83)	casamino acids ^g^	0.7
Y836 ^f^	13.4 (0.67)	1.56 (0.24)	none	8.6
**Does Y836 have intrinsic mutator activity? Transduce Y836 ( *cIII-ren*)λ into 594 and W3101.**				
594::(*cIII-ren*)λ	2.21 (0.48)	0.178 (0.085)	none	12.4
W3101::(*cIII-ren*)λ	11.9 (4.6)	1.50 (0.11)	none	7.9
**Assay requirement of SOS gene products for dual RK^−^ plus auxotrophic mutation(s).**				
Y836 *his lexA*3[Ind^−^]	0.26	0.011	histidine	23.6
Y836 *his* Δ*recA*	4.43 (0.43)	0.405 (0.062)	histidine	10.9
Y836 *his umuC*:Tn*5*	21.3 (0.96)	0.268 (0.032)	histidine	79.5
Y836 *his dinB*:Kan	5.00 (0.85)	0.244 (0.11)	histidine	20.5
594::(*cIII-ren*)λ *dinB*:Kan	1.9	0.090	none	21.1

^a^ Frequency of RK^−^ mutants forming colonies at 42 °C per colony forming units (CFU) at 30 °C on RM or MM × 10^−6^. (SE × 10^−6^) is the standard error, determined for ≥3 independent assays; ^b^ Frequency of RK^−^ mutants forming CFU at 42 °C per CFU at 30 °C on MM (minimal medium, or where noted, MM with the supplement nutrient added shown in adjacent column) × 10^−6^ (standard error × 10^−6^); ^c^ The frequency of RK^−^ mutants forming CFU on RM/frequency of RK^−^ mutants forming CFU on MM, or where noted on MM+ supplement; ^d^ Average of 10 independent assays and (standard error); ^e^ Average of 6 independent assays and (standard error); ^f^ Average of 5 independent assays and (standard error); ^g^ Medium includes Bacto Casamino Acids (not “Bacto Vitamin-Free Casamino Acids”).

**Table 2:** Blocking λ gene expression and *ori*λ replication initiation, or only *ori*λ initiation.

RK^+^ Strains ^a^	CFU at 42 °C/CFU at 30 °C		Ratio CFU on RM/MM
	RM	MM+Histidine	
**Inhibiting λ fragment gene expression at 42 °C by *cI*^+^ expressed on plasmid pCI**			
Y836 *his* [pCI^+^]	0.95 (0.076)	0.85 (0.033)	1.1
W3101::(*cIII-ren*)λ [pCI^+^]	1.2	1.2	1.0
594: (*cIII-ren*)λ [pCI^+^]	0.92	0.90	1.0
**Inhibiting replication initiation at 42 °C from *ori*λ on λ fragment by host mutation**			
Y836 *his dnaB*grpD55	0.91 (0.084)	0.15 (0.073)	6.1
Y836 *his dnaB*grpD55 [pCI^+^]	0.77	0.82	0.9

^a^ The identical λ fragments in each of the RK^+^ strains carry a *cI*857 mutation conferring to the CI857 repressor a temperature sensitive phenotype. The pCI^+^ plasmid encodes a WT *cI*^+^ gene and the CI^+^ repressor remains active (*trans* dominant), preventing transcription from promoters *p_L_* and *p_R_* at 30 and 42 °C. Values in parentheses represent standard error for multiple assays.

**Table 3:** Stab assay screen for auxotrophic colony forming units (CFU).

Strains	Assayed CFU ^a^	Auxotrophs/CFU ^a^ (Ts CFU) ^a^
**Nonlysogens or lysogens with noninduced prophage**		
CFU from strains spread on RM, 30 °C		
594	551	0
594 *dnaB*grpD55 ^b^	413	0
Y836	2520	0
Y836 *dnaB*grpD55 ^b^	1179	0
594: (*cIII-cI*857*-O*^+^*-P*^+^*-ren*)λ	347	0
Y836 RK^−^ Bib11t *O*^+^ *P*:Kan ^c^	324	0
Y836 RK^−^ 566a *O*^+^ *P*:IS*2* ^d^	280	0
Y836 RK^−^ *O*208b *P*^+^^e^	322	0
Y836 RK^−^ *O*223a *P*^+^^f^	223	0
Y836 RK^−^ 534c *O*^+^ *P*^+^^g^	167	0
CFU from strains spread on RM, 42 °C		
594	260	0
594 *dnaB*grpD55 ^b^	919	1 ^h^
Y836 [pCI]	88	0
Y836 *dnaB*grpD55 [pCI]	100	0
594: (*cIII-cI*857*-O*^+^*-P*^+^*-ren*)λ [pCI]	110	0
Total	7803	1
**RK^−^ CFU isolated from Induced RK^+^ strains (each expressing *P* at 42 °C)**		
Y836	904	94 (79)
Y836 *dnaB*grpD55	433	14 (11)
Y836 Δ*recA*	259	36 (36)
Y836 *dinB*:Kan	100	8 (8)
594: (*cIII-cI*857*-O*^+^*-P*^+^*-ren*)λ RK^+^	427	57 (57)
**Characterized λ replication defective RK^−^ mutants (forming CFU at high viability at 42 °C)**		
Y836 RK^−^ Bib11t *O*^+^ *P*:Kan	716	0
Y836 RK^−^ 566a *O*^+^ *P*:IS*2*	643	0
Y836 RK^−^ *O*208b *P*^+^	1605	1 (1)
Y836 RK^−^ *O*223a *P*^+^	654	11 (11)
Y836 RK^−^ 534c *O*^+^ *P*^+^	875	1 (1)
Total	6607	222 (204)

^a^ Isolated CFU appearing on RM agar plates, that were incubated at 30 or 42 °C, were picked to RM plates that were incubated at 30 °C, or to parallel MM+histidine plates that were incubated both at 30 °C and 42 °C to distinguish acquired auxotrophs with a temperature sensitive (Ts) phenotype. Values in “( )” represent number of auxotrophic CFU with a Ts phenotype forming CFU on both RM and MM+His plates incubated at 30 °C, but showing no growth on MM+histidine plates incubated at 42 °C; ^b^ The grpD55 mutation in host gene *dnaB* prevents λ replication initiation, but does not noticeably influence the growth of the *E. coli* host carrying the mutation; ^c^ SH insertion, clone Bib11t of kanamycin resistance (promoter-orf-terminator of gene *aph* from TN*903* removed from plasmid p51kan [76]) gene into *P* by recombineering, substituting bp 39651-39838 of *P*. The Kan insertion includes a synthetic 11 bp all-translational stop sequence at each end of the insertion; ^d^ The RK^−^ 566a mutation *P*:IS*2* represents an insertion in P right of 39690 with 6 bp repeat of bases 39685-39690 AGGTAG –IS*2*–AGGTAG. Blast of about 1000 bp sequence downstream of 39690 shows that the insertion is IS*2*; however, sequencing from both ends shows a 6 bp (not characteristic 5 bp) repeat sequence straddling each end of the insertion. The sequence of *oop-O* is wild type; ^e^ The RK^−^ mutation *O*208b is a 7 bp deletion in *O* (bases 39147-39153, fusing bases 39146-39154) creating −1 frameshift, with stop codon at 21 codons downstream from fused codon; ^f^ The RK^−^ mutation *O*223a is a 44bp deletion in *O* (bases 39044-39087, fusing ITN1 with ITN3) creating −1 frameshift, with stop codon 33 codons downstream from fused codon; ^g^ The RK^−^ mutant 534c was shown to have a wild type λ sequence from gene *cI*857 through *cII-oop-O-P-ren* through base 40713 in *ninB*. The strain likely survives replicative killing at 42 °C via an uncharacterized host mutation that prevents replication initiation from *ori*λ within the λ DNA fragment in strain Y836.

**Table 4:** Influence of *P* expression on the selection of rifampicin-resistant (Rif^R^) CFU.

**Strains with Fragment of λ Prophage**	**Frequency Rif^R^ CFU at 42 °C/30 °C ^a^ [Average Frequency Rif^R^ CFU at 30 °C]**
594	1 [8.3 × 10−8]
Exp. A. ^b^	
Y836 *O*^+^ *P*^+^	88,727 (42,027) [4.33 × 10−8]
Y836 *O*^+^ *P*^+^ *dnaB*grpD55	3 (1.5) [1.38 × 10−7]
Y836 *O*^+^ *P*:Kan	6 (1.9) [5.3 × 10−8]
Y836 *O*^+^ *P*:IS*2*	3 (0.24) [2.83 × 10−8]
Y836 *O*223a *P*^+^	2167 (730) [8.5 × 10−8]
Exp. B. ^b^	
Y836 *O*^+^ *P*^+^	42,500 (17,000) [5.4 × 10−8]
Y836 *O*^+^ *P*^+^ *dnaB*grpD55	2 [1.3 × 10−8]
594::(*cIII-cI*857*-O*^+^*-P*^+^*-ren*)λ	51,485 [1.0 × 10−7]
Y836 *O*^+^ *P*^+^ [pcI]	1 [2.4 × 10−8]
Y836 *O*^+^ *P*^+^ *dnaB*grpD55 [pCI]	1 [1.0 × 10−9]
594::(*cIII-cI*857*-O*^+^*-P*^+^*-ren*)λ [pCI]	1 [5.0 × 10−9]
**Strains with a Plasmid**	**Frequency Rif^R^ CFU at 37 °C/30 °C [Average Frequency Rif^R^ CFU at 30 °C] ^e^**
594 [pcIpR-*P*-timm]	122 (52) [3.0 × 10−8]
594 [pcIpR-*P*^π39991^-timm] ^c^	2 [3.3 × 10−8]
594 [pcIpR- *P*^∆76^-timm] ^d^	3 [4.3 × 10−8]
594 *dnaB*grpD55 [pcIpR-*P*-timm]	2 (1.1) [1.3 × 10−8]
594 *dnaB*grpD55 [pcIpR-*P*^π^-timm]	4 [3.5 × 10−8]
594 *dnaB*grpD55 [pcIpR-Δ*P*-timm]	0.1 (0.05) [2.0 × 10−9]

^a^ Averaged results for three to seven experiments (standard error). Other results show average determinations; ^b^ Experiments A and B were undertaken by different workers; ^c^ The mutation *P*^π39991^ is G to A transition, R137Q in *P*; ^d^ The mutation *P*^∆76^ is an in frame 76 codon deletion fusing part of codon 9 with 86, and deleting λ bases 39609-39836 in NH_2_ terminal end of *P*; ^e^ Determination of the frequency of Rif^R^ CFU was measured for multiple assays at 25 °C, 30 °C and 37 °C (the results per temperature is an average). The reduced level of *P* expression was measured at 37 °C from the pcIpR-*P*-timm plasmid [35]. The frequencies of Rif^R^ CFU selected at 25 °C and 30 °C were close and only the results for 30 °C are shown.

**Table 5:** Isolation and characterization of Rif^R^ CFU from 40 fluctuation assay (FA #1) tubes ^a^.

40 FA #1 Tubes	Rif^R^ CFU/Spread Plate		Sequence of One (or Multiple) ^d^ Rif^R^ CFU from	
Inoculate 15 CFU/mL, Grow 48 h at 25 °C	Spread 0.1 mL to Rif^100^ Plate, Incubate 72 h at 25 °C ^b,c^	Spread 0.1 mL to Rif^100^ Plate, Incubate 72 h at 37 °C ^b,c^	Rif^100^ Plate Incubated at 25 °C	Rif^100^ Plate Incubated at 37 °C
A4	3 ^b^	0	1585:CtoT, R529C	none
A6	1 ^b^	0	1595:CtoA, A532E	none
A7	5 ^b^	1	1527:CtoA, S509R	lost
A9	1 ^b^	4	1687:AtoC, T563P	lost
A10	1 ^b^	6 ^c^	1592:CtoT, S531F	1547:AtoG, D516G
B1	6 ^b^	2 ^c^	1605-13: ΔAGGCGGTCT, PGGL535-538P	443:AtoC, Q148P
B8	0	2 ^c^	none	1712:TtoA, L571Q (2) ^d^
B10	8 ^b^	0	1600:GtoT, G534C	none
C1	0	2 ^c^	none	1691:CtoT, P564L (2)
C4	4 ^b^	1 ^c^	1687:AtoC, T563P	1319-24:ΔGCGAAG, GEV440-442V (2)
C5	3 ^b^	4 ^c^	1691:CtoT, P564L	1691:CtoT,P564L
C6	11 ^b^	1 ^c^	1592:CtoT, S531F	1351:CtoA, R451S (2)
C7	1	9 ^b^	lost	1601:GtoA, G534D (3)
C10	1 ^b^	0	1586:GtoA, R529H	none
D1	1 ^b^	1 ^c^	1574:CtoG, T525R	1714:AtoC, I572L (3)
D2	2 ^b^	1 ^c^	1576:CtoG, H526D	1351:CtoA, R451S (2)
D3	0	7 ^c^	none	436:GtoT, V146F (2)
D5	1 ^b^	0	1527:CtoA, S509R	none
D6	6 ^b^	2 ^c^	1601:GtoT, G534V	1609:GtoT, G537C
D7	0	2	none	lost
D8	2 ^b^	0	1691:CtoT, P564L	none
D9	1 ^b^	1	1565:CtoT, S522F	lost
D10	1 ^b^	0	1604-12: ΔCAGGCGGTC, PGGL535-538P	none

^a^ Forty culture tubes with one mL RM broth, numbered A1–A10, B1–B10, C1–C10 and D1–D10, were inoculated with ~15 CFU (determined by parallel titration of the inoculum) of fresh 594[pcIpR-*P*-timm] culture, single colony 3. The tubes from which no Rif^R^ CFU were obtained are omitted in the left column, e.g., A1–A3, A5, *etc.* All the inoculated tubes were shaken in a water bath at 25 °C for 48 h. Thereupon, 0.1 mL aliquots, representing about ~2 × 10^8^ CFU were spread on two RM agar plates containing 100 µg/mL rifampicin. One plate was incubated at 25 °C and the other at 37 °C and the CFU arising are shown in columns 1 and 2, respectively; ^b^ Isolated, restreaked clone(s) retained the plasmid pcIpR-*P*-timm during growth in culture tube from 15 CFU/mL to ~2 × 10^9^ CFU/mL, as evidenced by efficient growth on Amp^50^; RM agar plates; ^c^ Isolated, restreaked clone(s) had lost plasmid pcIpR-*P*-timm during growth in culture tube from 15 CFU/mL to ~2 × 10^9^ CFU per mL; ^d^ Individual clones sequenced shown in parentheses.

**Table 6:** Transformation (×10^−7^) of pcIpR-*P*-timm/100 ng plasmid into Rif^R^ mutants of 594 **^a^**.

Strains/Mutants	Transformants at 25 °C	Transformants at 37 °C
594	520	<0.17
**594 Rif^R^ mutants selected from cells never exposed to gene *P*^b^**		
1-25A2	7900	<0.18
3-25E	570	<0.17
1-37A2	17,000	<0.19
3-37D	270	<0.18
T-3-37-B10	580	<0.2
T-3-37-C5	1200	<0.2
T-3-25-D10	780	<0.3
Td-3-25-A7,scg31	2800	<0.23
Td-3-37-C7,sc1f45	1200	<0.3
Td-3-25-C10	3800	1.2
Td-3-25-D9	1000	2.4
**594 Rif^R^ mutants selected at 37 °C from 594[pcIpR-*P*-timm] cultures that had lost the plasmid ^c^**		
3-37-A10	270	<0.3
3-37-B1	410	6.0
3-37-B8	1200	430
3-37-C1, 3-37-C5 ^d^	180	<0.12
3-37-C4	860	140
3-37-D1	100	<0.17
3-37-C6, 3-37-D2 ^e^	1100	390
3-37-D3	190	<0.2
3-37-D6	630	13

^a^ Rif^R^ isolates were streaked for sc’s on fresh LB agar plates with 50 µg/mL rifampicin, A CFU was selected, inoculated into LB broth and grown overnight to saturation. These cells were used for transformation with pcIpR-*P*-timm as described in Materials and Methods. Values with “<” had no recovered Amp^R^-transformants and the frequency shown was obtained by dividing “1” by the cell titer on LB agar plates. The results for one experiment are shown, but are representative of numerous repeats; ^b^ Refer to Table S2 for isolates employed; ^c^ Refer to Table 5 for isolates employed; ^d^ Both mutants with Rif^R^ mutation at 1691:CtoT, P564L, were P^S^, data for 3-37-C1; ^e^ Both mutants with Rif^R^ mutation at 1351:CtoA, R451S, were P^R^, data for 3-37-D2.

**Table 7:** pcIpR-*P*-timm∆*rop* transformation (× 10^−7^) into Tet^S^ *rpoB* Rif^R^ mutants and their transductants made Tet^R^ *rpoB* ^+^ Rif^S^.

Strains/Mutants/Phenotype	Transformants at 25 °C	Transformants at 37 °C
594 (Tet^S^ Rif^S^ *rpoB* ^+^)	800	<6.3 × 10^−9^
594 Tet^S^ *rpoB* Rif^R^ mutants		
Rif^R^ 3-37-C4	1500	110
Rif^R^ 3-37-D2	90	80
Rif^R^ 3-37-D6	1100	700
594 Rif^R^ mutants transduced to Tet^R^ *rpoB* ^+^ Rif^S^		
Tet^R^ Rif^S^ 3-37-C4	300	<1.3 × 10^−9^
Tet^R^ Rif^S^ 3-37-D2	1800	<2.3 × 10^−9^
Tet^R^ Rif^S^ 3-37-D6	600	<2.8 × 10^−9^

^a^ Cells were transformed with 1710 ng (in 2 µL) of pcIpR-*P*-timm∆*rop* plasmid.

**Table 8:** Bacteria and plasmids employed.

**Bacterial Strains**	**Characteristics or Genotype**	**Source/Ref.’; Hayes Lab # ^a^**
594	F^−^ *lac*-3350 *galK*2 *galT*22 *rpsL* 179 IN(*rrnD-rrnE*)1; also called R594	[71,92] SH lab; B10
594 *dnaB*-*grpD55*	*grpD55* allele *malF*3089:Tn*10*; Tet^R^, λ^R^ at 42 °C, λ*rep*P22^S^	[71,93] NB295
594 *lexA*3[Ind^−^] *malB*:Tn*9*	LexA repressor induction defective	[35] C. Erker (CE), NB293
594 Δ(*srlR-recA*)306:Tn*10*	deletion of *recA* Tet^R^ UV^S^	[35] CE, B318
594:*nadA*:Tn*10* [~*cIII*-*ren*]^λ^	Tn*10* [zbh29 at 16.8 min] *bio^+^* transductant = 594 *bio275* (λ*cIII*-*cI*[Ts]857-O-P-*ren*) Δ431	[73] = AC; this strain = NY1057
W3101: *nadA*:Tn*10* [~*cIII*-*ren*]^λ^	Tn*10* [zbh29 at 16.8 min] *bio^+^* transductant = 594 *bio275* (λ*cIII*-*cI*[Ts]857-O-P-*ren*) Δ431	AC, NY1057
CAG12147=*nad*A57:Tn*10* at 16.85 min	λ^−^, *nadA57*:Tn*10*, *rph*-1	C.A. Gross (CAG); NY1053
CAG12164	*malF*:Tn*10*, 4,241,898 [94]	Coli genetic stock center cgsc.biology.yale.edu
CAG12185	*argE*:Tn*10*, 4,151,734 [94]	cgsc.biology.yale.edu
CAG18500	*thiC*:Tn*10*, 4,192,143 [94]	cgsc.biology.yale.edu
Y836	SA500(λ*bio*275*cI*[Ts]857 Δ431) *his^−^*	[68,69] SH, NY1049
Y836 [pCI^+^]	λ prophage fragment genes rendered noninducible by WT CI^+^	[71]
Y836*his*^+^	*his^+^* transductant of Y836 *his* = Y836	AC, NY1046
Y836 RK^−^ *P*:kan (Bib11t)	SA500 (λ*bio*275 *cI*[Ts]857 *O^+^P*:kan Δ431) *his^−^* Kan^R^	[35] SH, NY1153
Y836 RK^−^ ilr566a	*O*^+^ *P*:*IS*2	CH, MY843 RK^−^
Y836 *dnaB*grpD55[pCI]	λ prophage fragment genes rendered noninducible by WT CI^+^	[71] AC, NY1054
Y836:*Tn*10	*nad*A57:Tn*10* at ~16.85 min CG12147	AC, NY1047
594::(*cIII-ren)*^λ^	*nad*A57:Tn*10* and *bio*275 = *Bio*^+^ (λ*cIII-cI[*Ts*]857-ren*) Δ431 transduced from Y836:*Tn*10	AC, NY1057
594::(*cIII-ren* ilr566a)^λ^	*nad*A57:Tn*10* and [*bio*275 λ*cIII-cI[*Ts*]857-ren*) Δ431 transduced from Y836 RK^−^ ilr566a *nad*A57:Tn*10*	AC, NY1065
594::(*cIII-ren*)^λ^ [pCI]	λ prophage fragment genes rendered noninducible by WT CI^+^	AC, NY1055
W3101	*gal*T22 IN(*rrnD*-*rrnE*)	B. Backmann, B25-b
W3101::(*cIII-ren*)^λ^	*nad*A57:Tn*10*	AC, NY1051
W3101::(*cIII-ren*)^λ^[pCI^+^]	λ prophage fragment genes rendered noninducible by WT CI^+^	AC, NY1059
SF2006 and SF2139	*dinB*:Kan	M. Goodman,
GW3200	AB1157 *umuD*_44_	D. Ennis, B396
GW2100	AB1157 *umuC*112:Tn*5* (Kan)	G. Walker, B317
Y836 *dinB*:Kan	*dinB*:Kan from SF2006	AC, 1045
Y836 *umuC*112:Tn*5*	*umuC*112:Tn*5* (Kan)	AC, 1052
Y836 ∆*recA* Tet^R^ UV^S^	∆(*srlR-recA*)306::Tn*10* [35]	AC, Y916, NY1048
Y836 *lexA*3[Ind^−^] *malB*:Tn*9*	LexA repressor induction defective	AC, from DE407 [35]
**Plasmids**	**Transformed into strain 594**	**Source/Ref.’; Hayes lab # ^a^**
pCI^+^	used to place WT CI^+^ repressor expression in strains to prevent λ prophage fragment derepression upon culture shift from 30 to 42 °C	M. Horbay; [71]
pcIpR-D-CAP-timm	D-CAP expression	CH, P459 [95]
pcIpR-*P*-timm	*Bam*HI-*Cla*I fragment from λ*cI*857, replacing D-CAP in P459, with λ bp’s 39582-40280	CH, P466 [35]
pcIpR-*P*-timmΔ*rop*	P651 = 4371bp; Δ *rop* region, *i.e.*, 613bp between *Pci*I to *Eag*I and includes 23937-24310bpλ) from pSJH-D[pR-MT-GFP-timm][EGFP]+λSO = P593, was inserted into P466 replacing its *rop* region between bp 938-2472	CH, P593; KR P651
pSIM6	inducible λ Red genes	L. Thomason [96,97]

^a^ The strain numbers are from Hayes laboratory collections. All gene inserts within the plasmids were sequenced to confirm genetic integrity of the inserted fragment.

## Supplementary Materials

The following are available online at http://www.mdpi.com/1999-4915/8/6/172/s1, Figure S1. pcIpR-P-timmΔrop transformation and retention in RifR isolates and transductants. Table S1: Plating efficiency of RK+ strains on RM and MM at 30 °C. Table S2: Spontaneous RifR mutations obtained without cell exposure to P. Table S3: 107 RifR mutations localized to rpoB. Table S4: Oligonucleotides employed for PCR fragment amplification and DNA sequencing.
