# Supplementary material for: Lambda gpP-DnaB Helicase Sequestration and gpP-RpoB Associated Effects: On Screens for Auxotrophs, Selection for RifR, Toxicity, Mutagenicity, Plasmid Curing
Source: Viruses. 2016 Jun 22;8(6):172. doi: 10.3390/v8060172 (PMC4926192; doi:10.3390/v8060172)
Supplement: Supplementary file 1 [file viruses-08-00172-s001.pdf]

Supplementary Materials: Lambda gp*P*-DnaB Helicase Sequestration and gp*P*-RpoB Associated Effects: On Screens for Auxotrophs, Selection for Rif^R, Toxicity, Mutagenicity, Plasmid Curing

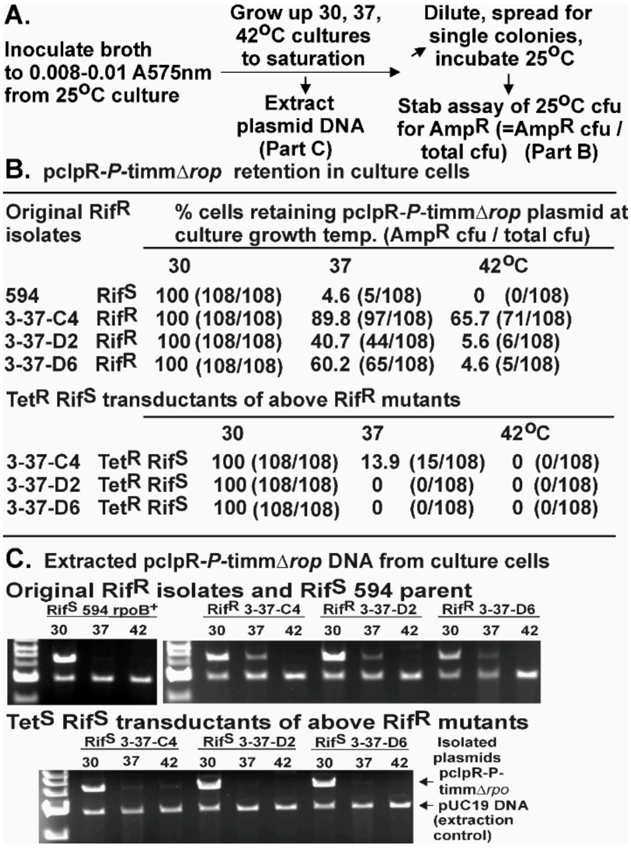

Figure S1. pcIpR-P-timmΔrop transformation and retention in RifR isolates and transductants. The experiments parallel those in Figure 5 undertaken with pcIpR-P-timm. For explanation of sections A-C, refer to legend for Figure 5.

Table S1. Plating efficiency of RK$^+$ strains on RM and MM at 30 °C.

RK⁺ Strains	*Titer × 10⁹ on RM* [a]	*Titer × 10⁹ on MM* [a]	*Cell Titer on MM/RM*
Y836 *his*	1.07 (0.23)	1.13 (0.15)	1.1
Y836 [b]	1.36 (0.08)	1.4 (0.06)	1.0
594	0.9 (0.17)	1.0 (0.17)	1.1
594:*λcIII-ren*	1.18 (0.12)	1.12 (0.11)	0.95
W3101	0.7	0.8	1.1
W3101:*λcIII-ren*	0.2	0.2	1.0

[a] TB agar represents “rich” medium, RM. MM is minimal medium, which for Y836 *his* mutant assays (top line) was supplemented with histidine. The values in parentheses represent standard error based on 3 to 5 independent assays; [b] His$^+$ transductant of Y836 *his*.

Table S2. Spontaneous Rif^{R} mutations obtained without cell exposure to P.

**Original Mutant Designation**	**Sequence of Rif^{R} CFU(s)**
1-25A2 [a]	1535:CtoA, S512Y
1-37A2 [a]	1586:GtoA, R529H
3-25E [a]	1592:CtoT, S531F
3-37D [a]	1714:AtoT, I572F
**Electroporation into 594 cells of PCR fragment containing midway the *original mutation*** [b]	
3-25-B10 1600:GtoT	T-3-25-B10 1607:GtoT, G536V
3-37-C5 1691:CtoT, P564L	T-3-37-C5 1532:TtoG, L511R
3-37-D10 1604-12:ΔCAGGCGGTC	T-3-37-D10 1604-12: ΔCAGGCGGTC
**Recovered mutant(s)s from P1vir transduction of *original mutant* into 594** [c]	
3-25-A7 1527:CtoA	Td-3-25-A7 representative CFU's:<br>443:AtoT, Q148L; 1342:CtoA, L448I
3-37-B8 1712:TtoA	Td-3-37-B8 1592:CtoT, S531F<br>Td-3-37-B8 1691:CtoT, P564L
3-37-C4 1319-24:ΔGCGAAG	Td-3-37-C4 representative CFU's:<br>1527:CtoG, S509R; 1527:CtoA, S509R<br>1538:AtoC, Q513P; 1547:AtoT, D516V<br>1586:GtoA, R529H; 1592:CtoT, S531F<br>1691:CtoT, P564L
3-37-C7 1601:GtoA	Td-3-37-C7 representative CFU's:<br>1532:TtoG, L511R; 433:AtoT, I145F
3-25-C10 1586:GtoA	Td-3-25-C10 443: AtoT, Q148L
3-25-D9 1565:CtoT	Td-3-25-D9 representative CFU's:<br>1525:AtoC, S509R<br>1600:GtoA, G534S

[a] Four culture tubes with one mL RM broth were inoculated with ~15 CFU of fresh 594 culture cells. The tubes were shaken in a water bath at 25 °C for 48 h. Thereupon, 0.1 mL aliquots, representing about ~2 × 10^{8} CFU were spread on two RM agar plates containing 100 ug/mL rifampicin and incubated at 25 or 37 °C, yielding mutants 3-25 and 3-37. Mutants 594-1-25 and 594-1-37 were obtained by simply spreading cells from a 30 °C overnight culture of 594 on RM RIF100 plates that were incubated at 25 or 37 °C and picking Rif^{R} CFU; [b] 594 cells were transformed with pSIM6. The PCR fragments used for sequence analysis were electroporated into 594 [pSIM6] cells and the cells were spread on SOB agar plates containing 100 ug/mL rifampicin. Rif^{R} CFU were isolated and the *rpoB* gene was sequenced; [c] The single clones (sc's) of the original Rif^{R} mutants (see Table 7) were grown in RM broth and two successive P1vir lysates were prepared on each clone. 594 culture cells were transduced with the 2° P1 lysate, and Rif^{R} CFU, seemingly representing P1 transductants, were isolated on LB agar plates containing 100 ug/mL of rifampicin (RIF100 plates). The *rpoB* gene in several single CFU (sc's) of the "transduced" clones were sequenced and the mutation conferring rifampicin resistance in each "transductant" is compared to the original Rif^{R} mutation for each clone.

Table S3. 107 Rif^R mutations localized to *rpoB*.

RpoB (Rif^R) Mutation	Base Change & [Source]
N139K	[1]
R143W	[1]
V144W	[1]
I145P	[1]
I145F	433:A to T #, ##
V146W	[1]
V146F	436:G to T # [2]
V146G	437:A to C [3]
Q148K	442:C to T [3] [a]
Q148R	443:A to G [3]
Q148L	443:A to T # [3] P^R
Q148P	443:A to C # [3,4] P^R
Q148H	444:G to T [3]
Q148H	444:G to C [3]
R151S	[4]
P153L	[4]
G181V	[4]
Y395D	[4]
L420R	[4]
ΔGEV 440–442 V	Δ1319-24 #, ## P^R
H447P	[4]
H447R	[4]
L448I	1342:C to A # [4]
R451S	1351:C to A #, ## P^R
G507D	A to G [3]
S508P	[3]
S509R	1525:G to T # [3]
S509R	1527:A to C # [3] P^R
L511R	1532:T to G #, ##
L511R	A to C [3]
L511P	A to G [3]
L511Q	A to T [3]
S512P	A to G [3]
S512Y	1535:G to T # [3]
S512F	A to G [3]
S512A	A to C [3]
Q513R	A to G [3,5]
Q513L	A to T [3,6,7]
Q513P	1538:A to C # [3,8,9]
Q513K	G to T [3]
D516V	1547:A to T # [2,3,10,11]
D516G	1547:A to G # [2,3,10,12]
D516N	G to A [3]
D516A	A to C [3]
D516Y	G to T [3]
N518D	A to G [3]
S522F	1565:G to A # [7]
S522Y	G to T [3]
E523V	A to T [3]
T525R	1574:G to C # [3]
H526D	1576:G to C # [3]
H526Y	G to A [3]
H526L	A to T [3]
H526R	A to G [3,5]
H526P	A to C [3,5]
H526N	G to T [3]
H526Q	G to T [3]
H526Q	G to C [3,5]
R529C	1585:G to A # [3,7]
R529H	1586:G to A # [3]
R529L	G to T [3]
R 529S	G to T [3,7]
Δ	Δ1589-97 [11]
S531F	1592:G to A # [2,3,10,11]
A532E	G to T [3,4]
A532E	1595:C to A #, ##
A532V	G to A [3,13]
L533P	A to G [3]
L533H	A to T [3]
L533R	A to C [3]
G534C	1600:G to T # [3]
G534S	G to T [3]
G534S	1600:G to A #, ##
G534V	1601:G to T # [3]
G534D	1601 :G to T # [3]
G534A	G to C [3]
ΔPGGL 535-538P	Δ1604-1612 #, ##
ΔPGGL 535-538P	Δ1605-1613 #, ##
P535L	[13]
G536V	1607:G to T # [4]
G537C	1609:G to T #, ## P^R
G537D	[4]
G544D	[9]
F545S	[9]
V550E	[4]
H551P	[4]
P560L	[12]
T563P	1687:A to C # [3,4,14]
T563I	[13]
P564L	1691:C to T # [3]
P564R	1691:G to C [3]
P564S	[12]
E565A	[12]
G570C	G to T [3]
L571Q	1712:T to A # [4] P^R
I572L	1714:A to C # [3]
I572F	1714:A to T # [3,7]
I572T	A to G [3]
I572N	A to T [3]
I572S	A to C [3,4]
I572M	G to C [3]
S574F	G to T [3]
S574Y	G to T [3]
R637C	[12]
H673Y	[12]
R687H	G to A [14]
S788F	[4]
G1260	[4]
H1244Q	[4]

[#]–This paper. ## This paper, unique *rpoB* mutation. P^R–mutation confers P-resistant phenotype to cells. [a] The mutation Q148K reported [3] as 442: G to T, was revised to be Q148K, 442: C to T.

Table S4. Oligonucleotides employed for PCR fragment amplification and DNA sequencing.

rpoB Primer Name	5′ to 3′ Sequence
L-rpoB298+20	ctgcgtctggtgatctatgagc
L-rpoB1421+20	cggtgaaagagcgtctgtctc'
L-rpoB1391+19	tccgcgttggcctggtacgt
L-rpoB321+20	cgaagcgccggaaggcaccgt
L-rpoB1391+19	tccgcgttggcctggtacgt
L-RpoB+ends FWD Set 1	tgactactgctgtgcctttc
L-RpoB+ends FWD Set 2	aacggtactgagcgtgttatc
L-RpoB+ends FWD Set 5	cggcccatatatctctgaaacc
L-RpoB mid-COOH end FWD Set	cccgatcgaagatatgccttac
L-RpoB mid-COOH end FWD Set	ctgctatcgaagaaggcaacta
L-RpoB mid-COOH end FWD Set	tcaccaccatccacattcag
L-RpoB+COOH end FWD Set 2	gatcaacgccatgctgaaac
L-RpoB+COOH end FWD Set 4	ggtatcggcgacaagatcaa
R-RrpoB752-19	gcggtttcaccacgcaggcg
R-rpoB1263-22	gctcaggataccggaaccttcga
R-rpoB2140-21	caccggagtcaacggcaacagc
R-rpoB2168-23	acaccaccacgtttagctaccgca
R-poB+ends REV Set1	gaaccacggtaagggatgatac
R-rpoB+ends REV Set 2	gataccggaaccttcgatttct
R-rpoB+ends REV Set5	ggcaagttaccaggtcttctac
R-rpoB mid-COOH end REV Set 1	tgacctgtttgagcgagaatta
R-rpoB mid-COOH end REV Set 2	cacttccgcaccaatgtaaac
R-rpoB mid-COOH end REV Set 4	ataccttttcgcagccatacc
R-rpoB+COOH end REV Set 2	acttcaccgaaagaccatgaa
R-rpoB+COOH end REV Set 4	ccgtcggagttagcacaat
DnaA-1	acgaccacctaacggacc
DnaA-2	gtacgtgagctggaaggg
DnaA-3	cccttccagctcacgtac
DnaA-4	cggataaccctggcggt
DnaA-5	accgccagggttatccg
DnaA-6	gcagggtcttttcgacgt
λ or plasmid primer name	sequence
L18	ttgccggaagcgaggcc
L21	cgcaacagtaaccagcat
L22	tgctgcttgctgttcttg
LMH29	ctgctcttgtgttaatgg
LMH32	cacagatctatagcaaac
L38985p20	gcagcaaggcggcat gtttgg
R9+1	tggtcagaggattcgcc
R17	taagactccgcatccgg
RPG2	aatgactcctgttgatag
RPG6	caatcgagccatgtcgtc
RMH25	ctgctcacggtcaaagtt
RMH33	gcgacgtccccaggtaat
R39280m21	ctgcggcggtcaggtcttct gc
R40769m22	gctgcggttgcgttcctgaa tgg
R1536-19	gaagacagtcataagtgcgg

References

1. Severinov, K.; Soushko, M.; Goldfarb, A.; Nikiforov, V. Rif^R mutations in the beginning of the *Escherichia coli rpoB* gene. *Mol. Gen. Genet. MGG* **1994**, *244*, 120–126.
2. Lisitsyn, N.A.; Sverdlov, E.D.; Moiseyeva, E.P.; Danilevskaya, O.N.; Nikiforov, V.G., Mutation to rifampicin resistance at the beginning of the RNA polymerase beta subunit gene in *Escherichia coli*. *Mol. Gen. Genet. MGG* **1984**, *196*, 173–174.
3. Garibyan, L.; Huang, T.; Kim, M.; Wolff, E.; Nguyen, A.; Nguyen, T.; Diep, A.; Hu, K.; Iverson, A.; Yang, H.; *et al*. Use of the *rpoB* gene to determine the specificity of base substitution mutations on the *Escherichia coli* chromosome. *DNA Repair* **2003**, *2*, 593–608.
4. Trautinger, B.W.; Lloyd, R.G. Modulation of DNA repair by mutations flanking the DNA channel through RNA polymerase. *EMBO J.* **2002**, *21*, 6944–6953.
5. Severinov, K.; Soushko, M.; Goldfarb, A.; Nikiforov, V. Rifampicin region revisited. New rifampicin-resistant and streptolydigin-resistant mutants in the beta subunit of *Escherichia coli* RNA polymerase. *J. Boil. Chem.* **1993**, *268*, 14820–14825.
6. Das, A.; Merril, C.; Adhya, S. Interaction of RNA polymerase and rho in transcription termination: Coupled ATPase. *Proc. Natl. Acad. Sci. USA* **1978**, *75*, 4828–4832.
7. Jin, J.; Gross, C.A. 3-Rpobc Mutations That Suppress the Termination Defects of Rho Mutants Also Affect the Functions of Nusa Mutants. *Mol. Gen. Genet.* **1989**, *216*, 269–275.
8. Guarente, L.P.; Beckwith, J. Mutant RNA polymerase of *Escherichia coli* terminates transcription in strains making defective rho factor. *Proc. Natl. Acad. Sci. USA* **1978**, *75*, 294–297.
9. Lisitsyn, N.A.; Sverdlov, E.D. Moiseveva, E.P.; Nikiforov, V.G. Localization of mutation leading to resistance of E. Coli RNA polymerase to the antibiotic streptolydigin in the gene *rpoB* coding for the beta-subunit of the enzyme. *Bioorg. Khim.* **1985**, *11*, 132–134.
10. Ovchinnikov Yu, A.; Monastyrskaya, G.S.; Gubanov, V.V.; Lipkin, V.M.; Sverdlov, E.D.; Kiver, I.F.; Bass, I.A.; Mindlin, S.Z.; Danilevskaya, O.N.; Khesin, R.B. Primary structure of *Escherichia coli* RNA polymerase nucleotide substitution in the beta subunit gene of the rifampicin resistant rpoB255 mutant. *Mol. Gen. Genet. MGG* **1981**, *184*, 536–538.
11. Ovchinnikov, Y.A.; Monastyrskaya, G.S.; Guriev, S.O.; Kalinina, N.F.; Sverdlov, E.D.; Gragerov, I.; Bass, I.A.; Kiver, I.F.; Moiseyeva, E.P.; Igumnov, V.N.; *et al*. RNA polymerase rifampicin resistance mutations in *Escherichia coli*: Sequence changes and dominance. *Mol. Gen. Genet. MGG* **1983**, *190*, 344–348.
12. Brandis, G.; Wrande, M.; Liljas, L.; Hughes, D. Fitness-compensatory mutations in rifampicin- resistant RNA polymerase. *Mol. Microbiol.* **2012**, *85*, 142–151.
13. Landick, R.; Stewart, J.; Lee, D.N. Amino acid changes in conserved regions of the beta-subunit of *Escherichia coli* RNA polymerase alter transcription pausing and termination. *Genes Dev.* **1990**, *4*, 1623–1636.
14. Jin, D.J.; Gross, C.A. Mapping and sequencing of mutations in the *Escherichia coli rpoB* gene that lead to rifampicin resistance. *J. Mol. Biol.* **1988**, *202*, 45–58.

© 2016 by the authors. Submitted for possible open access publication under the terms and conditions of the Creative Commons Attribution (CC-BY) license (http://creativecommons.org/licenses/by/4.0/).
